# Climatic implications on variations of Qehan Lake in the arid regions of Inner Mongolia during the recent five decades

**DOI:** 10.1007/s10661-016-5721-5

**Published:** 2016-12-13

**Authors:** Xi Chun, Riguge Su, Jiyao Liu, Wenjun Liang, Mei Yong, Khkhuudei Ulambadrakh

**Affiliations:** 1 Inner Mongolia Key Laboratory of Mongolian Plateau Environment and Global Change, Inner Mongolia Normal University, 81 Zhaowuda Road, Hohhot, Inner Mongolia 010022 China; 2 College of Life Science and Technology, Inner Mongolia Normal University, 81 Zhaowuda Road, Hohhot, Inner Mongolia 010022 China; 3 Inner Mongolia Repair Engineering Laboratory of Wetland Eco-environment system, Inner Mongolia Normal University, 81 Zhaowuda Road, Hohhot, Inner Mongolia 010022 China; 4 Department of Geology and Geophysics, National University of Mongolia, Sukhbaatar district 46A/#481, Ulaanbaatar, Mongolia

**Keywords:** Semi-arid environment, Lake level fluctuation, Inner Mongolia, Climatic change, Wetlands

## Abstract

The Qehan Lake Basin (QLB) and its system of lakes are located in a marginal monsoon zone and are extremely sensitive to global climate change. In this paper, using aerial photographs from different periods, in addition to MSS, TM, and ETM images, and combining these with regional topographic maps, we analyze lake area changes from 1958 to 2010 and the relation between Qehan Lake (QL) and climate variability. Our results indicate that there was a relatively high lake level in 1959, when the area and volume of the lake were 118.9 km^2^ and 151.9 × 10^6^ m^3^, respectively, but this level was subject to a shrinking trend until 2010, when the lake area was only 28.1 km^2^, and the water volume was 41.1 × 10^6^ m^3^. West Qehan Lake (WQL) has experienced severe water shrinkage and lake level fluctuation. In 1958, WQL was 80.2 km^2^ in area and 124.1 × 10^6^ m^3^ in volume. However, due to a rapid decrease in precipitation and increases in both temperature and evaporation, it began to dry up in 2002. The WQL Water area decreased by 1.82 km^2^/a, and the lake level declined by 7 m during 1958–2002, so it became an ephemeral lake.

## Introduction

Terminal lakes are extremely sensitive to climate change in marginal monsoon zones. Lake area variations (Poianik et al. 1996; Ma et al. 2010; Zhu et al. 2010) and lake level fluctuations (Hartmann et al. 1990; Yu et al. 2003; Li et al. 2007; Wunnemann et al. 2012; Madsen et al. 2014) can accurately indicate climatic variations in the watershed between wet and dry periods.

Over the last five decades, in the arid region of northwest China, lake levels have tended to rise, accompanied by an expansion in lake area and an increase in runoff (Ma et al. 2008; Chen et al. 2009). Similar phenomena have occurred on the central Tibetan Plateau (Bian et al. 2009; Zhu et al. 2010), with the increasing precipitation in northwest China (Li et al. 2009; Liu et al. 2011) and the change in climate from warm-dry to warm-humid (Shi et al. 2007). Climate simulations also confirm that mean annual precipitation (MAP) has increased by 20% in northwest China in the twenty-first century as a result of global warming (Ding et al. 2007). However, the results of recent research show that precipitation in northwest China tended to decrease as a whole, but increased in particular areas, and drought had an increasing trend from 1961 to 2010 (Sun et al. 2013). Correspondingly, there are different degrees of atrophy in the lakes of the Tibetan Plateau where precipitation is the main runoff source (Morrill et al. 2004; Shao et al. 2008; Huang et al. 2011). Over the past three decades, 59 lakes have dried up and disappeared, and the water area has reduced significantly over the Inner Mongolian plateau within the marginal monsoon zone (Ma et al. 2010). On the Alxa Plateau, there has been a significant reduction in river runoff (Ma et al. 2008), the total oasis area has strongly atrophied, and eco-environmental problems have become more prominent, most especially in western Inner Mongolia. Even lakes in the arid region of central Asia have atrophied during 1975–2007 (Kezer and Matsuyama 2006; Bai et al. 2011). At the same time, the lakes of eastern and central Inner Mongolia, such as Hulun Lake, Dalinur Lake, and Daihai Lake (Niu et al. 2012; Wang et al. 2013), have atrophied at different rates in response to desertification and a decline in the wetland ecosystems surrounding the lakes. During the past decades, most rivers suffered a reduction in runoff, and some even dried up, explaining the gradual reduction in the number of lakes and lake areas in Inner Mongolia (Feng et al. 2011; Liang et al. 2011; Wang et al. 2011). Clearly, there are differing spatial and temporal parameters for understanding lake evolution of both the arid and cold regions of China.

Different understandings of the driving mechanisms behind lake level fluctuation are discussed in arid China and Mongolia. Some postulated that climate change leads to lake retreat (Li et al. 2007; Szumińska 2016), while others insist that inappropriate human economic activity is the main factor in lake area reduction (Zhao et al. 2005; Zheng et al. 2006; Tao et al. 2015). The mechanisms driving lake level fluctuations in marginal monsoon zones thus need to be further studied.

Over the past five decades, Qehan Lake (QL) not only experienced a severe contraction but also became an important source of strong dust storms affecting northern China. As such, QL provides a typical example of lake evolution in arid China. In this paper, combining field trips with remote sensing data from different periods, we analyze the changes in lake area and lake levels, establish a chronological framework for lake changes, rebuild lake evolution, and according to meteorological data, discuss the response of lakes in arid areas to global climate change.

## Materials and methods

### Study area

**Fig. 1.**
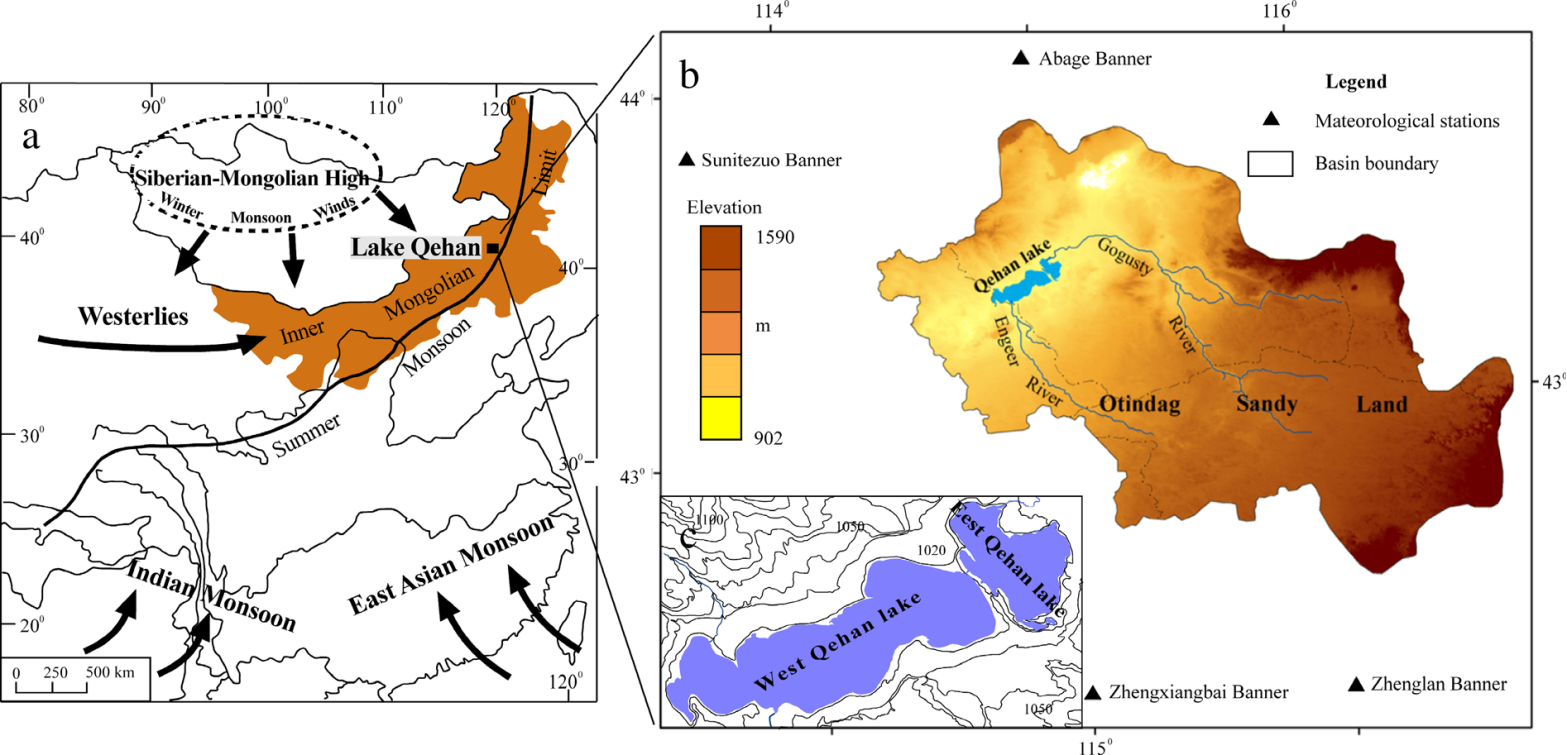
**a** Map of North China. **b** Qehan Lake (QL) catchment and location of four meteorological stations. **c** Qehan Lake area

The Qehan Lake Basin (QLB) formed in the early Tertiary. The area lies at an elevation of 900–1500 m asl and is located 80 km southwest of the Abaga Banner in the Xilingol League, Inner Mongolia (Fig. 1a). The Otindag Sandy Land lies to the south, the Abaga grassland to the north (Fig. 1b). The QLB includes parts of the Abaga, Zhenglan, Zhengxiangbai, and Sunitezuo banners of the Xilingol League within the Inner Mongolia Autonomous Region.

QL is divided into two parts by a 2 km long spit (the spit is a basis of the dam built in 2010). In the early 1950s, a 2 m wide sluice connecting the east Qehan Lake (EQL) and the west Qehan Lake (WQL) was built (Fig. 1c) on the spit. The area of EQL is 28 km^2^, with a maximum depth of 7.2 m. The area of WQL is 80 km^2^. The Gogusty and Engeer rivers flow to the north into EQL and WQL, respectively. The Gogusty River is 110 km long, with its annual runoff of 36.02 × 10^6^ m^3^ in the 1970s. The Engeer River is 80 km long, with a short, periodic flow and little water. Both of the rivers are seasonal.

The predominant climate is Asian Monsoon, accompanied by cold, dry winters and hot, rainy summers. Mean annual precipitation (MAP) ranges from 360 to 240 mm, about 70% of which falls in the rainy months of June–August. Mean month temperatures in the catchment range widely between −18.1 and 21.3 °C. In winter, the area is controlled by Mongolian high pressure, where temperature can reach as low as −42 °C, and snowfall always makes up a significant proportion of precipitation. The November–March period is freezing, and strong winds and cold temperatures are commonplace.

The QLB, a grassland region dedicated to traditional Mongolian animal husbandry, covers an area of 14,211 km^2^ and has a population of about 38,000 without irrigated agricultural activity in the watershed area. Main land cover types are grassland, sandy land, and forest. As QL is inland, far from the big cities, human activity is limited and has so little impact on lake level fluctuations until 2010. The eco-environment of the lake remains in a semi-natural state, so it is an ideal site to study climate and lake environment changes over the past decades.

### Data

The aerial photographs of QL used in this study were taken during1958–1959 and provided by the Inner Mongolian Key Laboratory of Mongolian Plateau Environment and Global Change. The multi-spectral scanning images (MSS) were downloaded from the global land cover facility of the University of Maryland (http://glcfapp.glcf.umd.edu). The Landsat TM and enhanced TM (ETM) remote sensing images were downloaded from the United States Geological Survey at http://glovis.usgs.gov *.* The scale 1:50,000 topographic map was used (Table 1). We used general meteorological data from four stations surrounding QL, located in Abaga, Zhenglan, Zhengxiangbai, and Sunitezuo banners (Fig. 1b). The data selected were those closely related to climate change, temperature, and precipitation (Table 2).

**Table 1.** Remote sensing date and materials in QLB

Materials	Date(s)	Range	Remarks
Aerial photographs	Jun. 18, 1958; Aug. 10, 1959	Lake area	Scale 1: 10,000
LandSat_MSS	Oct. 4, 1973; May 28, 1975; Jul. 25, 1977; Jun. 29, 1983	Watershed	Resolution 80 m
LandSat5_TM	Oct. 4, 1991; Sep. 24, 1993; Oct. 3, 1999; Oct. 21, 2000; Jun. 2, 2001; Aug. 16, 2002; Aug. 27, 2006; Aug. 6, 2007; Aug. 3, 2009; Jul. 5, 2010	Watershed	Resolution 30 m
LandSat7_ETM+	May 7, 2003; Aug. 13, 2004; Sep. 17, 2005; Aug. 24, 2008	Watershed	Resolution 30 m
Topographic maps	1969	Watershed	Scale 1: 50,000
Elevation map	2004	Watershed	Scale 1: 10,000

**Table 2.** Meteorological site information

Station	Location	Elevation (m)	Mean temperature (°C)	Mean precipitation (mm)	Period
Abaga Banner	44°01′N, 114°57′E	1126.1	1.29	240.5	1953–2010
Zhenglan Banner	42°18′N, 116°00′E	1301	2.30	357.9	1971–2010
Zhengxiangbai Banner	42°18′N, 115°00′E	1347.8	2.38	353.9	1971–2010
Sunidzuo Banner	43°52′N, 113°38′E	1036.7	3.09	191.4	1956–2010

### Digital elevation model

**Fig. 2.**
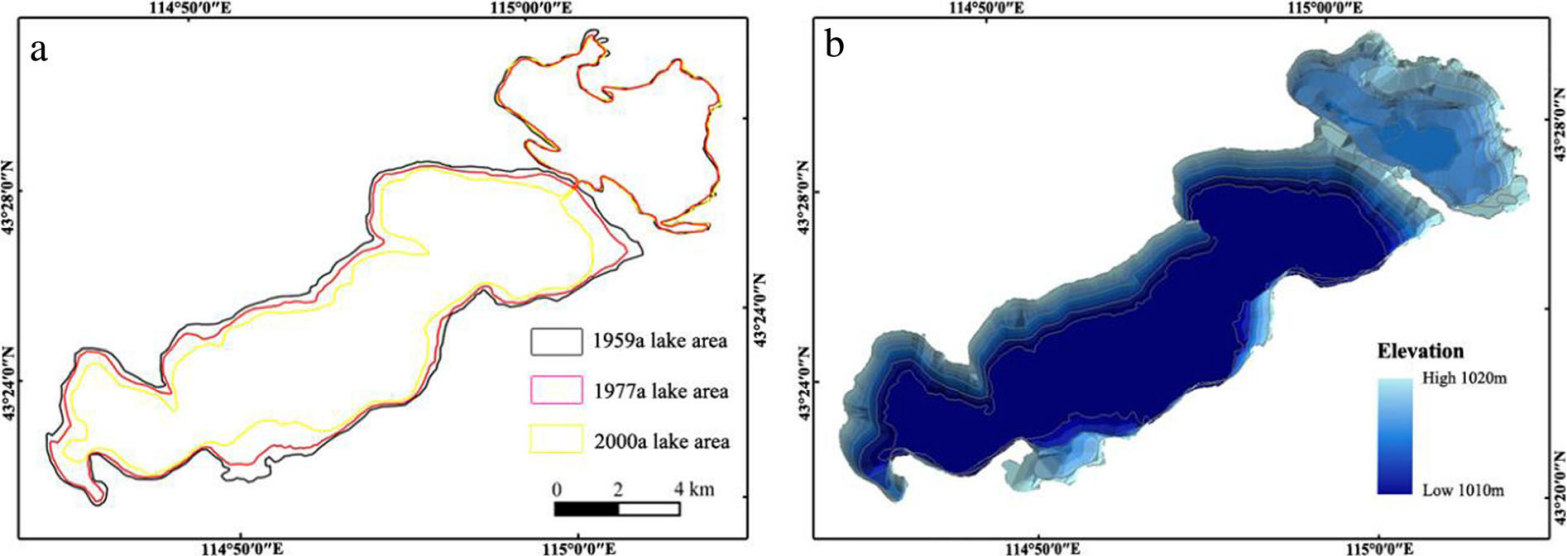
**a** Changes in the Qehan Lake (QL) area for different periods. **b** DEM diagram of lake depth

Using a 2008 ETM image as our base image, lower-resolution MSS images from 1973 as the base for our registration image, and the cubic convolution interpolation method, we resampled aerial photographs and MSS graphics to complete image registration. Fixed points were selected, such as buildings and spit tops. For geometric correction, classification, and precision testing, we used ENVI image processing software and used three band combinations to generate false color images. To highlight the remote sensing imagery of lake fluctuations, we used edge enhancement and a gray-scale transformation (Thomas et al. 2007; Ma et al. 2010). Then, using scale 1:10,000 and 1:50,000 topographic maps combined with field surveys, we generated elevation contour data digitally (Fig. 2a) and built a digital elevation model (DEM) of QL (Fig. 2b). By overlapping image maps with TM and ETM, we generated a DEM of lake depths at the ARCGIS interface and calculated lake depths, levels, and lake volume for the corresponding period.

### Mann–Kendall test

The Mann–Kendall nonparametric test (Mann 1945; Kendall 1975) has been widely used for the analysis of hydrological and meteorological chronologies and trends (Chen et al. 2009). Regression analysis was performed for analyzing the trends, and the sequential version of the Mann–Kendall test was used for the examination of their statistical significance. In the trend test, assuming that *H*_0_ represents the independent distribution of the *X* dataset, no trend is discernible. The optional hypothesis *H*_1_ indicates that the *X* dataset has a monotonic trend. The Mann–Kendall statistical test can be defined as follows:

1 $$ S={\displaystyle \sum_{t=1}^{n-1}{\displaystyle \sum_{k=t+1}^n\mathrm{s}\mathrm{g}\mathrm{n}\left({x}_k-{x}_t\right)}} $$

2 $$ \mathrm{s}\mathrm{g}\mathrm{n}\left({x}_j-{x}_k\right)=\left\{\begin{array}{c}\hfill +1\kern0.24em \mathrm{if}\kern0.24em \left({x}_j-{x}_k\right)>0\hfill \\ {}\hfill 0\kern0.24em \mathrm{if}\kern0.24em \left({x}_j-{x}_k\right)=0\hfill \\ {}\hfill -1\kern0.24em \mathrm{if}\kern0.24em \left({x}_j-{x}_k\right)<0\hfill \end{array}\right. $$

3 $$ Var(S)=\frac{\left[n\left(n-1\right)\left(2n+5\right)-{\displaystyle \sum_{i=1}^m{t}_i\left({t}_i-1\right)\left(2t+5\right)}\right]}{18} $$

4 $$ {Z}_c=\left\{\begin{array}{ccc}\hfill \frac{S-1}{\sqrt{Var(S)}}\hfill & \hfill \mathrm{if}\hfill & \hfill S>0\hfill \\ {}\hfill 0\hfill & \hfill \mathrm{if}\hfill & \hfill S=0\hfill \\ {}\hfill \frac{S-1}{\sqrt{Var(S)}}\hfill & \hfill \mathrm{if}\hfill & \hfill S<0\hfill \end{array}\right. $$

where *x*_*j*_ and *x*_*k*_ are the sequential data values, *n* is the dataset length, *t*_*i*_ is the number of ties of the extent *i*, and *Z* is the standardized test statistic value. *S* is the test statistic. When *x*_*j*_ − *x*_*k*_ is greater than, equal to, and less than 0, sgn(*x*_*j*_ − *x*_*k*_) is equal to 1, 0, and −1, respectively. If −*Z*_1−*α*/2_ ≤ *Z*_c_ ≤ *Z*_1−*α*/2_, one accepts the null hypothesis *H*_0_, where ±*Z*_1−*α*/2_ is a 1 − *α*/2 standard quantile and *α* is at a statistically significant level, when *α* = 0.05 and *Z*_1−*α*/2_ = ±1.96.

Monotonic trend test sequences can be used to determine the value of *β* as follows:

5 $$ \beta =\mathrm{Median}\left(\frac{x_{i^{-}\;}{x}_j}{i-j}\right)\kern0.72em \forall j<i $$

where *1* < *j* < *i* < *n* and *β* is the median of all the data series values in the adjacent cell. When *β* > 0, it reflects an upward trend, and when *β* < 0, it reflects a downward trend.

### Mann–Whitney test

Using time series of the watershed analyzes step changes by the Mann–Whitney test determining whether temperature and precipitation of every station existed in periodic variation and abrupt change processes. When time series reached an enough length of record(n ≥ 50), the general sample size is used to detect step changes in the regions (Xu et al. 2003). The data vector *X* = (*x*_1_, *x*_2_,…, *x*_n_), partition *X*, such that *Y* = (*x*_1_, *x*_2_,…, *x*_n1_) and Z = (*x*_n1+1_, *x*_n1+2_,…, *x*_n1+n2_). The Mann–Whitney test statistic is given as

6 $$ Zc=\frac{{\displaystyle \sum_{t=1}^{n_1}r({x}_t)}-{n}_1{n}_2\left({n}_1+{n}_2+1\right)/2}{{\displaystyle {\left[{n}_1{n}_2\left({n}_1+{n}_2+1\right)/12\right]}^{1/2}}} $$

In which *r* (*x*_*t*_) is the rank of the observations. The null hypothesis *H*_0_ is accepted if −*Z*_1−*α*/2_ ≤ *Z*_*c*_ ≤ *Z*_1−*α*/2_, where ±*Z*_1−*α*/2_ is a 1−α/2 quantile of the standard normal distribution corresponding to the given significance level *α* for the test.

## Results

### Abrupt change in temperature and precipitation

**Fig. 3.**
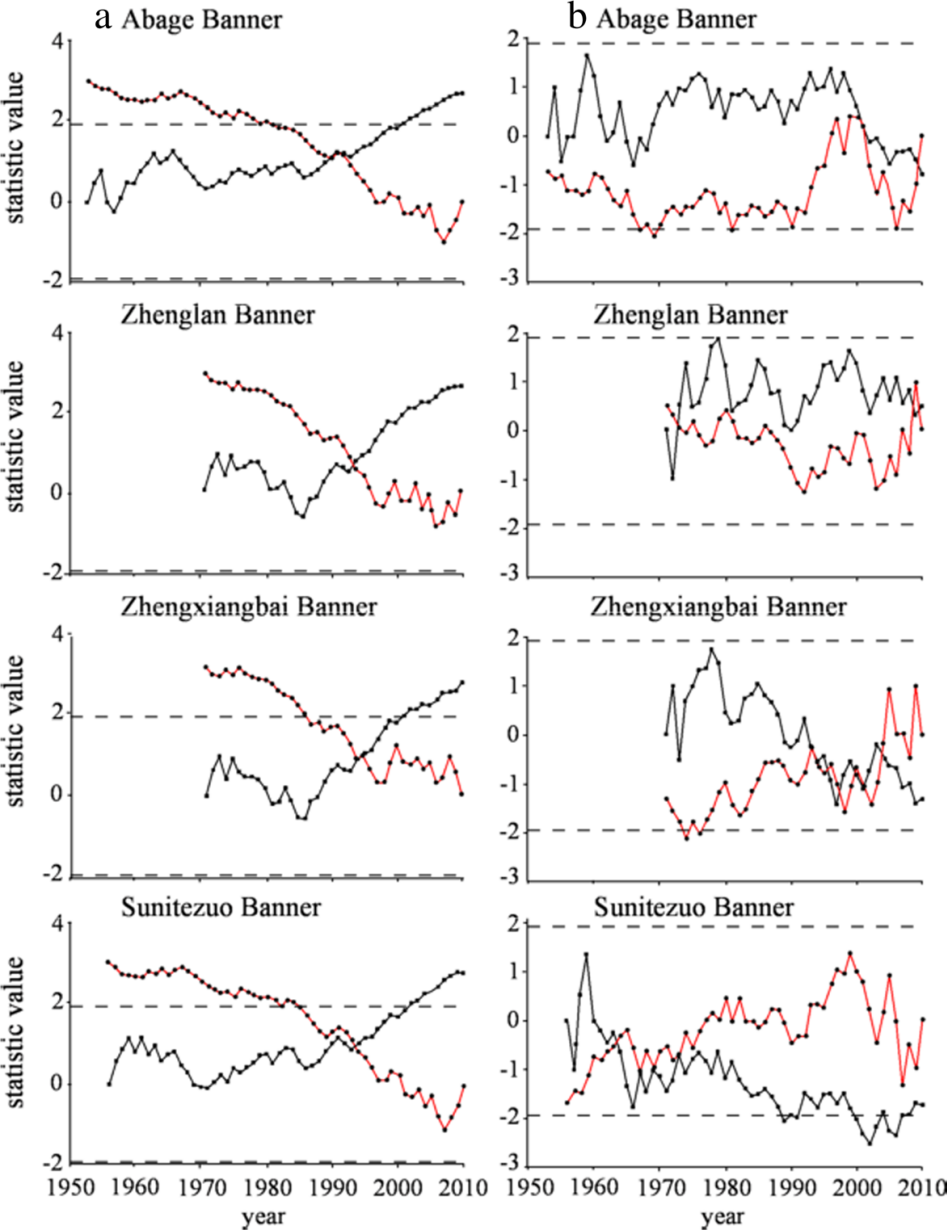
Sequential Mann–Kendall test for **a** annual temperature and **b** annual precipitation of each site

A Mann–Kendall test of each site indicated that temperature change points occurred in 1993 at the Abaga Banner Station and in 1994 at the other three sites (Fig. 3a). This showed that temperature change in the watershed became increasingly marked, with 1994 being the change point. The periods were separated as two parts, and the change points before and after the period were compared (Table 3); the results showed that the average temperature of the latter period was higher than the front by 1.15–1.81 °C, with an increase of 52–241.3%. Temperatures tended to rise significantly since 1994. The result of the Mann–Whitney test indicated that the time series of every station had a significant periodic variation process and reached a 0.01 level, which proved that the change points were 1993 and 1994 (Table 3).

**Table 3.** Mann-Kendall and Mann–Whitney test results of trend for temperature time series

Station	Change Point	Change point		Mann–Kendall test			Mann–Whitney test			
		*before*	*after*	*Z*_*C*_	*β*	*H*_*0*_	*n*_1_	*n*_2_	*Zc*	*H*_0_
Abaga Banner	1993	0.75	2.56	6.08***	0.05	R	40	18	5.12***	R
Zhenglan Banner	1994	1.81	3.23	4.73***	0.05	R	23	17	4.64***	R
Zhengxiangbai Banner	1994	1.97	3.28	4.88***	0.06	R	23	17	4.34***	R
Sunitezuo Banner	1994	2.69	4.09	5.26***	0.04	R	38	17	5.05***	R

*Zc*: The significance levels tested are 0.001 (***), 0.01 (**), and 0.05 (*). *β*: Positive (negative) value indicates an upward (downward) trend

*R* reject *H*_0_, *A* accept *H*_0_

The precipitation change point at the Abaga Banner Station occurred in 2001 (Fig. 3b). The Zhengxiangbai Banner Station possessed more than five change points in precipitation, but a significant change point occurred in 2001 (Fig. 3b). Though the intersection point did not occur at 2001 in the Sunitezuo Banner, the UB curve spanned a critical value of Z = −1.96 and could be viewed as occurring abrupt change in 2001, which reached significance at the 0.05 level. The three stations evinced a relatively concentrated period of precipitation, both at similar time. After their change point, precipitation at Abaga, Zhengxiangbai, and Sunitezuo banners dropped by 40, 19.7, and 13.3 mm, respectively, showing a 16.2, 5.5, and 6.8% decrease, respectively. Nevertheless, precipitation minimum values in the northern watershed occurred around 2001 and declined significantly. The result of the Mann–Whitney test demonstrated that the precipitation time series of Abaga and Zhenglan banner stations occurred in a significant downward trend around 2001 (Table 4).

**Table 4.** Mann–Kendall and Mann–Whitney test results of trend for precipitation time series

Station	Change point	Change point		Mann-Kendall test			Mann–Whitney test			
		*before*	*after*	*Z*_*C*_	*β*	*H*_*0*_	*n*_1_	*n*_2_	*Zc*	*H*_0_
Abaga Banner	2001	247.37	207.41	−0.76	−0.35	A	48	10	4.94***	R
Zhenglan Banner	2006	360.22	342.12	0.49	0.33	A	35	5	3.57***	R
Zhengxiangbai Banner	2001	358.91	339.19	−1.32	−1.20	A	30	10	1.56	A
Sunitezuo Banner	2001	193.84	180.48	−1.71	−0.86	A	45	10	4.91***	R

*Zc*: The significance levels tested are 0.001 (***), 0.01 (**), and 0.05 (*). *β*: Positive (negative) value indicates an upward (downward) trend

*R* reject *H*_0_, *A* accept *H*_0_

### Changing trends in temperature and precipitation

On an annual scale, the *Z*_*c*_ values for each site varied from 4.73 to 6.08, showing that temperatures had a significantly rising trend (Table 3). The *β* value for each site shows that the rate of the watershed temperature increase was 0.04–0.06 °C/a. On a seasonal scale, summer temperatures for each site showed a significant rising trend. In winter, apart from the Sunitezuo Banner Station, the rate of temperature at the other sites rose significantly.

The *Z*_*c*_ and *β* values for the sites (except for Zhenglan Station) are negative on an annual scale (Table 4), with a precipitation decreasing rate of 0.76–1.71 mm/a within the watershed. Seasonal precipitation for the Abaga and Sunitezuo banners (except spring) decreased over the whole watershed. The largest decline of precipitation occurred in summer, ranging from 0.24 to 1.37 mm/a.

### Changing trends in pan evaporation

In general, pan evaporation showed a slight increase in the watershed, with an increasing rate of 1.3–2.3 mm/a, but there were large fluctuations during the period study. For example, from 1955 to 1972, evaporation at the Abaga Banner Station was high, with an annual mean of 2020 mm. During 1973–1994, evaporation was low with an annual mean of 1880 mm and decreased by 6.9%. During 1995–2010, evaporation began to rise to 2085 mm, with an overall increase of 10.9%, most likely allied to increasing average temperatures.

### Lake evolution trends

The volume and area of WQL reached its maximum in 1959 and then dropped continuously, often fluctuating dramatically. From 1958 to 2002, 80 km^2^ of WQL dried up and 101.9 × 10^6^ m^3^ of water evaporated and disappeared, with a decreasing rate of 2.3 × 10^6^ m^3^/a. The lake level of WQL declined from 1016 to 1009 m. Conversely, the lake area, volume, and level of EQL increased by 3 km^2^, 10.2 × 10^6^ m^3^, and 0.2 m from 1958 to 2010, respectively.

## Discussions

### Lake change time series

#### Lake rapid expansion during 1958–1959

**Fig. 4.**
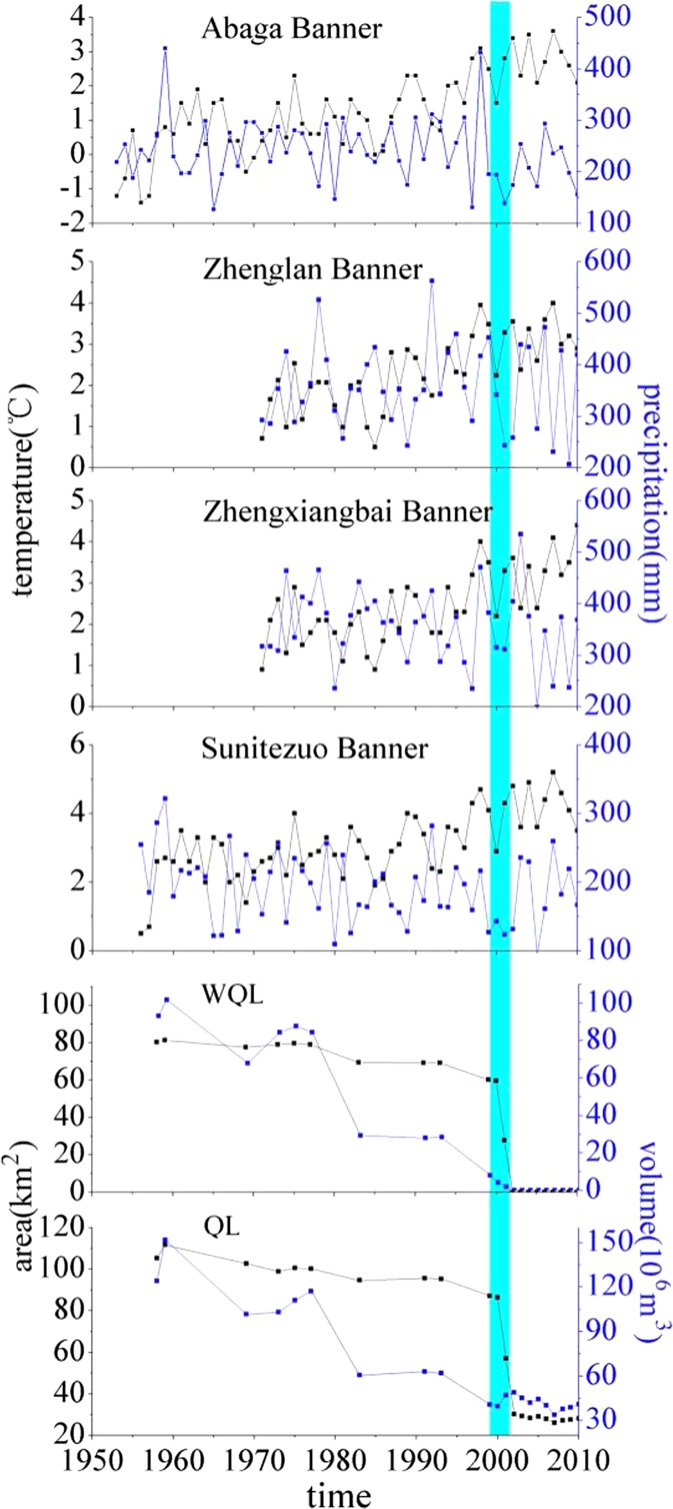
Site temperatures (*black line*) and precipitation values (*blue line*) versus trends in WQL and QL area (*black line*) and volume (*blue line*), 1958–2010

From 1958 to 1959, the rapid expansion of QL meant that it reached its peak level in the recent five decades, and its total area and volume reached 111.8 km^2^ and 151.9 × 10^6^ m^3^, respectively (Fig. 4). The total area of the lake increased by 6.5 km^2^, and the volume of the lake expanded by 27.8 × 10^6^ m^3^. The area of EQL and WQL increased by 5.4 and 1.1 km^2^, respectively, and their water levels rose to their peaks, accordingly. From 1953 to 1959, annual precipitation at the Abaga and Sunitezuo banner sites showed an upward trend overall: precipitation in 1959 attained its maximum value with the lake levels to peak, with the same maximum as area and volume. In summer of the same time, records showed that daily precipitation values for QLB were 38.8–98.8 mm, contributing to subsequent catastrophic flooding and peak lake inflow. In 1959, precipitation values reached their most recent peaks and exceeded mean annual rainfall rates by 191–614% in Inner Mongolia, especially at Daihai Lake, Dalinur Lake, and Huangqihai Lake in central Inner Mongolia. Other documents confirm that in the late 1950s, the rainy periods in arid regions were relatively wet (Zhai et al. 2005).

#### Lake shrinkage during 1959–1999

During 1959–1999, the WQL area decreased by 21.1 km^2^, with a reduction in lake water volume of 97.9 × 10^6^ m^3^ (Fig. 4) and a consequent 2.8 m drop in water levels. EQL experienced a gradual period of decline, with the total area and volume of lake water falling by 24.7 km^2^ and 111 × 10^6^ m^3^, respectively. During the same four decades, mean annual temperature (MAT) generally displayed an upward trend in QLB. For example, MAT in the watershed was 2.65 °C in the 1990s, which was higher by 2.03 °C than that in the 1950s. This warming trend became more significant after 1994. From this year onward, and accounting for its inter-annual variability, each site precipitation exhibited a decreasing trend (Fig. 4). For example, MAP in the 1990s was 15 mm less than in the 1950s. The combination of decreasing precipitation and rising temperature resulted in the reduced runoff into the lake, exacerbating the impact of increased lake water evaporation and reduction. Precipitation decreased significantly on the Mongolian Plateau in the 1960s and 1970s (Qian and Lin 2005). Despite precipitation which increased during the 1980s–1990s, the overall trend remained downward (Szumińska 2016). Relative humidity also fell steadily (Zhai et al. 2005), consistent with declining lake levels.

#### Lake rapid change during 1999–2002

In 1999, WQL was 60.2 km^2^ in the area (Fig. 4), but thereafter shrank rapidly to dry up completely in 2002. The former lakebed became dry salt crust. This indicated that the environmental dry event has happened. WQL was located in an arid zone, providing conditions favorable to water evaporation, and since the lake was shallow, the lake bottom was flat, and the gradient was small. Thus, lake evolution has a great response to climate change, as well as increasing temperature and evaporation and reducing precipitation. During 1999–2002, at Abaga and Sunitezuo banners, mean precipitation was only 176 and 132 mm, respectively, accounting for 73 and 69% of the MAP. Analysis of changes in precipitation has also revealed that variations occurred in Abaga, Zhengxiangbai, and Sunitezuo banners around 2001 (Fig. 3), confirming that precipitation reduced significantly around QL, which played an important role in the disappearance of WQL. During 1999–2002, the mean temperature of the watershed area reached 3.22 °C, which was higher than the MAT from the past five decades by 1.18 °C. Additionally, variations in regional temperatures increased significantly after 1994, leading to enhanced lake evaporation. Studies have shown that the temperature increased by 1 °C, with an increase in land surface evaporation of 5–6% (Philip and Biney 2002), leading to a severe drought. Thus, WQL dried up in 2002 (Fig. 4), which was not only the result of constant reductions in precipitation and water availability but was also due to rising temperature and increased lake surface evaporation. Records show that during 1999–2001, the Xilingol League suffered consecutive periods of drought of the longest duration of all the sites, with precipitation less than 50% of the MAP (Fan et al. 2009; Liu and Wang 2012). The area of desertification increased by 0.26 × 10^6^ km^2^, and the livestock number decreased by 6.844 × 10^6^ in Xilingol. Dalinur and Hulun lakes, near the QL, decreased and atrophied. The extreme drought extending from 1999 to 2002 occurred in northern China and Mongolia (Davi et al. 2006; Liu and Wang 2007).

#### Lake stability during 2003–2010

The EQL was about 28 km^2^ from 2003 to 2010. The greatest inter-annual fluctuation in the area was no more than 2 km^2^. The water level was relatively stable, varying between 0.1 and 0.2 m higher or lower than its annual means. Changes in temperature and precipitation during this period were slight.

### Relation between lake area and climate change

Lake fluctuation is closely related to climate change in arid areas (Li et al. 2007; Ma et al. 2007; Ma et al. 2010). Changes in temperature, precipitation, and pan evaporation were particularly prominent in the arid region of China (Qi et al. 2005). A positive correlation exists between the area of WQL and the precipitation measured at each site. The relation between the WQL area and precipitation values appears closest at the Abaga and Sunitezuo banner sites, where *R* ^2^ was 0.45 (*p* < 0.01) (*n* = 13, when sampled in 1958–2002). *R* ^2^ was 0.38 (*p* < 0.05) at the Zhenglan Banner station in the southern watershed. These values indicated that the meteorological stations located closer to the lake reflected changes in decreasing precipitation better over the lake and their consequent important fluctuations in lake levels.

Changes in the area of WQL exhibit a negative correlation with temperature, with *R* ^2^ ranging between 0.47 and 0.56. This correlation is highly significant (*p* < 0.01). It indicated that the spatial difference in temperatures was small and that temperature change was consistently upward. Therefore, the correlation between temperature change and lake area was clear.

Although evaporation exhibited a negative correlation with lake area, *R* ^2^ was only 0.03–0.17, demonstrating that the statistical relation was insignificant. So pan evaporation rates may be influenced not only by temperature, cloud cover, and wind speed but also by lake depth, area, shape, and local environment.

### Relation between lake volume and climate change

**Fig. 5.**
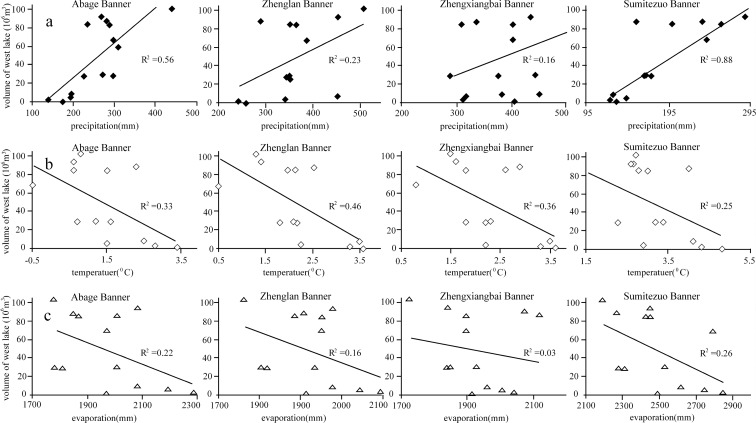
Relation between reductions in West Qehan Lake (WQL) water volumes. **a** Mean annual temperature. **b** Mean annual precipitation. **c** Mean annual evaporation, 1958–2002

The correlation between changes in WQL volume and precipitation for the Abaga and Sunitezuo banner sites was the closest, with *R* ^2^ of 0.56 (*p* < 0.01) and 0.88 (*p* < 0.01), respectively (Fig. 5a). The other two sites exhibited almost no correlation. Because the Abaga and Sunitezuo banner sites are near the lake, their datasets better reflected the actual precipitation received by the lake and its periphery. The two sites in the southern part of the Otindag Sandy Land are surrounded by high dune sand and are also 150 km from the lake. Therefore, they cannot accurately reflect precipitation and runoff values in the watershed and exhibit an ambiguous relation with lake volume. These findings showed that precipitation around the lake periphery is the main source of natural water recharge. In addition, the Engeer River is an inflow source for WQL, but the river blanked in the 1990s, meaning the lake lost a valuable recharge source. The Gogusty River flows into EQL, and historically, water has then been fed into WQL. So changes in WQL volume may reflect changes in precipitation around the watershed.

Nevertheless, the role played by temperature and evaporation changes in WQL volume cannot be ignored. The *R* ^2^ between temperature and lake water volume for the 1958–2002 period ranged between 0.25 and 0.46 (Fig. 5b). Moreover, the correlation between lake volume and temperature was clearer for the northern sites than for the southern sites. This may be related to the higher temperatures experienced in the south and may explain why the correlation for these southern sites was not statistically significant. Temperature exerted less impact on WQL volume than on WQL area. This would suggest that larger, shallower lakes respond more sensitively to rising temperatures.

There was no obvious relation between pan evaporation and WQL volume. *R* ^2^ was between 0.03 and 0.26 (Fig. 5c), which was statistically insignificant.

Fluctuations in lake volumes are the result of variations in temperature, precipitation, and evaporation (Li et al. 2007; Ma et al. 2008). We know from the disappearance of WQL that climate change can exert a profound impact upon lakes. In the study area, the climate exhibited a warming trend and was accompanied by significant reductions in MAP. The latter reinforced reductions in runoff and the consequent atrophying of WQL, and the former caused increases in lake surface evaporation rates, further accelerating the disappearance of the lake. Such a trend is consistent with the lake volume and area data collected over the past five decades. Variations in inter-annual lake volumes and area have been greatly influenced by precipitation values. As MAP reduced, MAT and lake surface evaporation rates rose, and lake volume and area decreased correspondingly. The time at which WQL finally dried up coincided with severe changes in MAP in the northern sector of the watershed. Precipitation has therefore played one of the most dominant roles in lake evolution.

## Conclusions

Over the past five decades, MAT of the watershed has gradually increased, rising at a rate of 0.49 °C/10a. This trend accelerated after 1994. MAP declined at a rate of 5.4 mm/10a. This downward trend became more marked in the northern part of the watershed region in 2001, and the warming and drying processes continued until 2010.

The QL area fell from 105.3 km^2^ in 1958 to 28.1 km^2^ in 2010, with a reduction of 73.3%. Volume reduced from 124.1 × 10^6^ m^3^ in 1958 to 41.1 × 10^6^ m^3^ in 2010, a reduction of 83 × 10^6^ m^3^. The ecological impact of this is evident in the reduction in wetlands, an expansion of desertification into areas of which was formerly lake, with an accompanying serious process of environmental degradation.

The shrinkage in lake area and volume has been caused by various factors, including decreasing MAP in the watershed and increasing MAT and evaporation rates. WQL has responded most sensitively to variations in precipitation, which has played one of the most dominant roles in lake fluctuations.

The watershed area suffered consecutive, severe droughts from 1999 to 2002. Marked changes in precipitation occurred in the north part of the watershed, with a significant decrease in MAP from 2001 onwards. Correspondingly, the area of WQL fell from 60.3 km^2^ in 1999 to nil in 2002, when it disappeared. These consecutive, severe periods of drought led to a marked decrease in precipitation over the watershed, and as temperatures rose and lake surface evaporation rates increased, the rate of drying up of WQL accelerated.
